# Novel Hypomorphic Mutation in* FANCD2* Gene Observed in a Fetus with Multiple Congenital Anomalies

**DOI:** 10.1155/2016/1462818

**Published:** 2016-08-23

**Authors:** Radoslava Vazharova, Svetlana Vragaleva, Violeta Dimitrova, Samuil Ivanov, Lubomir Balabanski, Maxim Malinov, Draga Toncheva

**Affiliations:** ^1^Gynecology and Assisted Reproduction Hospital “Malinov DM”, 1680 Sofia, Bulgaria; ^2^Department of Biology, Medical Genetics and Microbiology, Faculty of Medicine, Sofia University “St. Kliment Ohridski”, 1407 Sofia, Bulgaria; ^3^Medical Center “Femina”, 1202 Sofia, Bulgaria; ^4^University Ob/Gyn Hospital “Maichin Dom” and Department of Obstetrics and Gynecology, Medical University of Sofia, 1431 Sofia, Bulgaria; ^5^Department of Medical Genetics, Medical University of Sofia, 1431 Sofia, Bulgaria

## Abstract

Congenital anomalies affect 1% to 2% of the newborns. The urinary tract and the kidneys are involved in 4-5% of the cases while upper-extremities abnormalities are present in 10%. Certain anomalies occur in isolation, whereas others are associated with systemic conditions. The prenatal detection of fetal anomalies compatible with life is a challenge for both the parents and the physician. The prognosis for the fetus/newborn and the reproductive decisions of the family largely depend on the causes underlying the disease. The reported case is of a G2P1 pregnant woman referred for routine ultrasound scan at 24 weeks of gestation (w.g.). The fetus had growth retardation, right kidney agenesis, bilateral absence of radial bones and thumbs, radial deviation of the wrists, and short humeri. Nuchal fold thickness was 5 mm and there was a single umbilical artery. After termination of pregnancy, SNP array genotyping and next-generation sequencing of targeted candidate-genes were performed trying to clarify the etiology of the fetal polymalformative syndrome. A new hypomorphic mutation in* FANCD2* gene was found to underlie this fetal anomaly. The case illustrates that patients/families affected by rare monogenic disorders may benefit from application of modern technologies like microarrays and NGS.

## 1. Introduction

Major congenital malformations are reported in at least 2% of all fetuses and infants, having significant impact on mortality and morbidity in the perinatal period and during infancy and childhood [1]. With the advance of ultrasound technology in recent decades, an increasing number of congenital fetal malformations have become amenable to prenatal diagnosis. Ultrasound assessment of fetal anatomy between 19 and 23 weeks of gestation (w.g.) has become a routinely recommended procedure allowing the detection of about 64% of the severe congenital malformations [2]. One of the aims of prenatal diagnosis is to ensure optimal pregnancy management regarding antenatal care, referral for delivery to an adequate level medical setting, and planning of the postnatal treatment of the baby. In many countries, parents may opt for pregnancy termination if one or more severe disabling fetal malformations are diagnosed prenatally.

The prenatal detection of fetal anomaly/anomalies compatible with life is a challenge for both the parents and the physician. Clarifying the etiology may help in providing reliable information to the couple about the short and long term prognosis in the particular case. It may also have impact on parental decisions regarding current pregnancy and future reproductive choices.

New technologies like chromosomal microarray analysis and next-generation sequencing of gene panels/exomes/genomes make etiologic diagnosis possible in at least 10–15% of cases with structural anomalies [3, 4]. Combination of homozygosity mapping using SNP arrays and sequencing of target regions has been successfully applied postnatally to identify mutations causing certain autosomal recessive diseases. Herein we report on clinical and novel findings of a hypomorphic mutation in the* FANCD2* gene in a fetus with prenatally detected malformations of the upper limbs and the kidneys and the utility of SNP genotyping and next-generation sequencing (NGS) methods in such cases.

## 2. Case Presentation

A 29-year-old pregnant woman, G2P1, was referred at 24+4 weeks of gestation (w.g.) according to the LMP for routine fetal anomaly scan. Her past medical and family history were uneventful, with previous normal pregnancy that ended with the delivery of a healthy child. Ultrasound examination in the first trimester, performed at another institution, did not reveal fetal malformations and the combined screening test (NT and serum PAPP-A and free beta hCG) showed low risk for fetal trisomies 13, 18, and 21, monosomy X, and triploidy.

A detailed ultrasound examination at 24 w.g. demonstrated a male fetus with intrauterine growth retardation (IUGR); individual biometric parameters and the average ultrasound age (AUA) corresponded to 21 w.g. Bilateral upper limb anomalies were present including bilateral radial aplasia, absence of thumbs, and radial deviation of the wrists, accompanied by bilateral humeral hypoplasia (humeri corresponding to 19 w.g.). There was right kidney agenesia/aplasia and single umbilical artery. The nuchal fold thickness was 5 mm. The other fetal structures appeared normal as well as the amount of the amniotic fluid. Though debilitating, none of the observed abnormalities was incompatible with life.

The patient was referred for genetic counseling. Invasive prenatal diagnosis was proposed in order to clarify the etiology of malformations, to make a reliable prognosis for the fetus and to estimate recurrent risks in the family. However, the parents opted for termination of pregnancy and for genetic studies of the fetus after that.

All biological samples were collected after obtaining written informed consent from the parents. Genomic DNA from the aborted fetus was extracted from the umbilical cord after pregnancy termination and DNA from both parents was extracted from blood leucocytes using a commercial kit (Qiagen).

**Figure 1:**
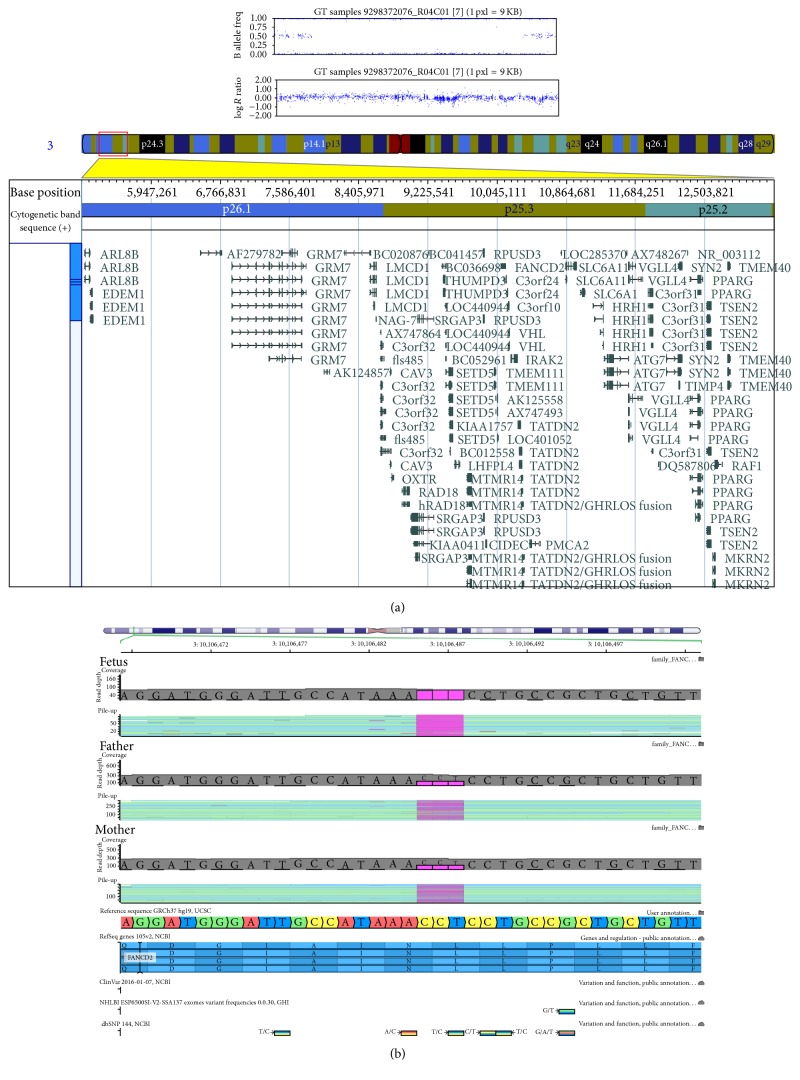
(a) Detailed view of the region with AOH in 3p: arr[hg19] 3p26.1p25.3p25.2(6206901-12352468)x2hmz. (b) The DNA sequences for exon 23 of the* FANCD2* gene of the fetus and both parents, visualized with Golden Helix GenomeBrowse 2.0.2 software. The deletion is marked in purple. The DNA sequences of the fetus and parents are shown aligned to the reference human genome sequence (GRCh37 hg19, UCSC) and corresponding amino acid sequence of the FNCD2 protein. The deletion is not reported in dbSNP 144 NCBI and NHLBI Exome Sequencing Project data tracks.

As first step in genetic analysis we applied SNP array genotyping of the fetal DNA with Illumina Human CoreExome-12. Compared to arrayCGH, SNP arrays show higher sensitivity for the detection of low-level mosaic aneuploidies and chimerism [5] and offer the ability to detect copy number neutral regions of absence of heterozygosity (AOH) [6]. The location of homozygous regions may be used for mapping information in families to identify autosomal recessive disease-causing genes. Consanguinity can be revealed by AOH, because multiple regions of AOH are expected to be present in individuals from inbred populations, representing chromosomal segments that are identical by descent after transmission through parental lineages. The test revealed no pathological deletions/duplications but a homozygous chromosome region encompassing around 6.1 Mb in 3p: arr[hg19] 3p26.1p25.3p25.2(6206901-12352468)x2hmz (Figure 1(a)). A total of 52 genes have been mapped to the region* (GRM7; LMCD1; NAG-7; C3orf32; CAV3; OXTR; RAD18; SRGAP3; THUMPD3; LOC440944; SETD5; LHFPL4; MTMR14; CPNE9; BRPF1; OGG1; CAMK1; TADA3; ARPC4; TTLL3; RPUSD3; CIDEC; JAGN1; IL17RE; IL17RC; CRELD1; PRRT3; TMEM111; LOC401052; CICE; FANCD2; C3orf24; C3orf10; VHL; IRAK2; TATDN2; C3orf42; GHRL; TATDN2/GHRLOS fusion; SEC13; ATP2B2; MIR885; LOC285370; SLC6A11; SLC6A1; HRH1; ATG7; VGLL4; C3orf31; SYN2; TIMP4; *and* PPARG)*, 13 of them are associated with monogenic human diseases and 3 of them* (CIDEC, JAGN1,* and* FANCD2)* are implicated in autosomal recessive diseases. The absence of heterozygosity observed in the fetus could lead to a manifestation of a recessive disease due to a combination of pathological mutant alleles in a homozygous state. The gene* FANCD2* located in the AOH region was nominated as a good candidate to explain fetal malformations in the particular case. As a second step of genetic analysis we conducted next-generation sequencing using the TruSight Cancer gene panel (Illumina), which apart from* FANCD2* includes all other known genes associated with Fanconi anemia. The fetus was found to be homozygous carrier of a novel hypomorphic mutation in the* FANCD2* gene: g.10106488_10106490delCCT, NM_033084.3: c.2097_2099delCCT, NP_149075.2: p.Leu700del. The mutation represents a deletion of three nucleotides in the sequence of exon 23 of the gene. This deletion preserves the reading frame but leads to a loss of a conserved leucine at position 700 in the protein product. The variant has not been observed among the 60000 individuals studied by the ExAC consortium, so it is not a common polymorphism. As hypomorphic mutations in* FANCD2* have been reported in patients with severe phenotype, we hypothesize that this variant is pathogenic and causative for the fetal malformations in this family. Both parents were found to be heterozygous carriers of the mutation (Figure 1(b)) and segregation of the variant in the family is in agreement with expected autosomal recessive mode of inheritance of the disease. Both parents of the fetus are of Bulgarian descent and their families come from two small villages in Montana district (in northwestern Bulgaria), so a distant relatedness between parents could not be excluded.

Pathogenic variants of the gene* FANCD2* are expressed phenotypically as Fanconi anemia (FA) type D2. The main features of the disease include developmental abnormalities in different organ systems (including radial ray defect, absent kidney, and growth retardation with prenatal onset), early-onset bone marrow failure (pancytopenia), and a high predisposition to cancer. The cellular hallmark of FA is hypersensitivity to DNA cross-linking agents and high frequency of chromosomal aberrations pointing to a defect in DNA repair. In this case we did not have the opportunity to check for chromosomal breakage in the presence of mitomycin C or diepoxybutane, because there were no viable fetal cells available for testing. Fanconi anemia type D2 (FA-D2) occurs in 3–6% of all patients with the disorder. Malformations are frequent in patients with FA-D2, and hematologic manifestations occur earlier and progress more rapidly than in any other patients with anemia of Fanconi [7].

## 3. Discussion

Fetal structural anomalies may be isolated or associated with systemic/syndromic conditions, so differential diagnosis may be challenging.

Upper limbs reduction defects account for around 10% of all prenatally detected congenital malformations, while unilateral kidney agenesia/aplasia accounts for 4-5%. Two extensive 11-year-long population studies suggest the prevalence of upper limb anomalies to be approximately 20 in 10,000 live births [8, 9]. Unilateral renal agenesis, defined as unilateral absence of kidney structure, has an estimated worldwide prevalence of around 1 in 2000 births [10]. As in daily clinical practice renal aplasia cannot be easily differentiated, the term unilateral renal agenesis is generally applied for both clinical entities.

Reduction anomalies/deformities of the upper limbs may be observed in many genetic disorders (chromosomal and monogenic) but may also result from teratogenic exposure during pregnancy [11]. Unilateral renal agenesis is highly heterogeneous as well [12]. A combination of renal and upper limb malformations, similar to what we described in our case, may be part of more than 20 syndromes, with different etiology including monogenic dominant and recessive diseases (Baller-Gerold syndrome, TAR syndrome, Townes-Brocks syndrome, acrofacial dysostosis 1, Nager type, Fanconi anemia, etc.), unknown sporadic conditions (VATER/VACTERL association), and microstructural chromosomal anomalies (10q24 trisomy, inv dup(22)(q11), etc.).

Our case illustrates the feasibility of new technologies in the genetic evaluation of patients with multiple malformations. SNP genotyping helps not only to screen the fetal genome for pathogenic copy number variants (including common aneuploidies and microdeletions/microduplications) but also to find out chromosome regions harboring genes implicated in monogenic systemic malformations. The prognosis for the developing fetus and the reproductive decisions of the family largely depend on the causes underlying the particular malformation. In our case, finding out the genetic etiology of the fetal phenotype made it possible to provide adequate genetic counseling to the family and to plan optimal disease prophylaxis in future pregnancies.
